# Longitudinal profiling of antiphospholipid antibodies reveals pre-conception levels as a key predictor of live birth in women with recurrent pregnancy loss

**DOI:** 10.3389/fimmu.2026.1765672

**Published:** 2026-05-29

**Authors:** Peng-Dan Luo, Qian Li, Si-Qi Wei, Peng-Sheng Zheng

**Affiliations:** 1 Department of Reproductive Medicine, The First Affiliated Hospital of Xi’an Jiaotong University, Xi’an, Shaanxi, China; 2 Department of Clinical Immunology, Xijing Hospital, the Fourth Military Medical University, Xi’an, Shaanxi, China

**Keywords:** antiphospholipid antibodies, dynamic changes, pre-conception screening, pregnancy outcomes, recurrent pregnancy loss

## Abstract

**Introduction:**

Studies indicate that approximately 15–20% of patients with recurrent pregnancy loss (RPL) test positive for antiphospholipid antibodies (aPL). However, the dynamic changes in aPL from pre-conception to early pregnancy in the RPL population and their relationship with pregnancy outcomes remain unclear. This study aimed to longitudinally analyze the dynamic changes of aPL profiles in RPL patients and evaluate their predictive value for pregnancy outcomes.

**Methods:**

100 patients diagnosed with RPL between March 2022 and December 2023 were enrolled. Serum levels of anticardiolipin antibody (aCL) and anti-β_2_-glycoprotein I antibody (aβ_2_GPI) isotypes (IgA, IgM, and IgG) were measured via chemiluminescence immunoassay at three timepoints: pre-conception, post-conception, and 3 months post-conception, with subsequent pregnancy outcomes tracked.

**Results:**

A total of 61 patients (61%) tested positive for at least one aPL subtype at one or more timepoints; however, only one met the 2006 Sydney classification criteria for antiphospholipid syndrome. Longitudinal analysis revealed that post-conception levels of aβ_2_GPI-IgM, aCL-IgA, and aCL-IgG were significantly elevated compared to pre-conception levels (all *p* < 0.05). Regarding outcomes, the live birth rate was significantly lower in pre-conception aPL-positive patients compared to those who were negative (44.8% vs 66.2%, *p* = 0.048), while the early pregnancy loss rate was significantly higher (51.7% vs 26.8%, *p* = 0.017). Multivariate logistic regression analysis identified three independent risk factors for subsequent pregnancy loss: higher number of previous pregnancy losses (OR = 1.871, 95% CI: 1.102–3.175, *p* = 0.020), elevated post-conception aCL-IgG level (OR = 1.336, 95% CI: 1.039–1.718, *p* = 0.024), and higher number of pre-conception aPL types positive (OR = 2.730, 95% CI: 1.039–7.173, *p* = 0.042). This model demonstrated fair discrimination with an area under the receiver operating characteristic curve (AUC) of 0.733 (95% CI: 0.631-0.835, *p* < 0.001).

**Conclusion:**

aPL levels increase significantly after conception in women with RPL and are independently associated with adverse pregnancy outcomes. Our findings suggest that aPL screening should be performed both before pregnancy and in early pregnancy in RPL patients to enable timely intervention and improve pregnancy outcomes. Pre-conception aPL testing may hold greater predictive value than in early pregnancy.

## 1 Introduction

The 2017 ESHRE guideline (1) defined recurrent pregnancy loss (RPL) as the loss of two or more pregnancies before 24 weeks of gestation. Studies have reported that the risk of one spontaneous abortion (SA) in women of reproductive age is approximately 10% (2), with an RPL incidence rate of 1–4% (3). Additionally, as the number of previous miscarriages increases in RPL patients, the risk of recurrence rises correspondingly. For patients with ≥3 consecutive miscarriages, the probability of recurrence reaches 40–80% (4). The etiology of RPL is complex and multifactorial. Currently, the four most significant causes are recognized as chromosomal abnormalities, anatomical uterine defects, autoimmune disorders, and endometrial dysfunction (3). Furthermore, over 50% of RPL cases remain unexplained, termed unexplained RPL (5). With advancements in modern reproductive immunology, increasing evidence suggested a close relationship between RPL and autoimmune antibodies. Research indicates that abnormalities in specific immune interactions at the maternal-fetal interface may be critical in disrupting normal pregnancy progression (6).

Antiphospholipid syndrome (APS) is a systemic autoimmune disorder characterized by the persistent presence of medium-to-high titer antiphospholipid antibodies (aPL) in circulation, clinically manifested by venous/arterial thrombosis and/or pathological pregnancy outcomes including recurrent early pregnancy loss, fetal growth restriction, intrauterine fetal demise, preeclampsia, and placental insufficiency (7). When pathological pregnancy is the primary clinical feature, it is termed obstetric APS (OAPS) (7). Currently, the diagnostic criteria for APS include three typical aPL: lupus anticoagulant (LA), anticardiolipin antibody (aCL), and anti-β_2_-glycoprotein I antibody (aβ_2_GPI).

As an autoimmune disorder, APS demonstrates significant association with RPL. Studies indicated that 15–20% of RPL patients test positive for aPL (3). The incidence of obstetric complications in APS patients reached as high as 38.6% (8). Major guidelines including RCOG (2011), ASRM (2012), and ESHRE (2017) all recommend aPL screening in RPL populations (1, 4, 9). Low-dose aspirin (LDA) combined with heparin remains the cornerstone therapy to improve live birth rates, with the consensus recommendations advocating for pre-conception initiation and sustained anticoagulation throughout pregnancy and postpartum (1, 4, 9). The precise mechanisms underlying APS-related adverse obstetric outcomes remain incompletely elucidated. Current hypotheses suggest that the harmful inflammatory effects of aPL on placental and endothelial cells lead to placental vascular thrombosis and placental dysfunction (10, 11). *In vitro* studies indicated that aPL-induced excessive complement activation correlated with poor pregnancy outcomes (12). Additionally, aPL may interfere with the proliferation and invasion of extravillous trophoblast (13).

Current evidence suggests that maternal aPL levels may correlate with different pregnancy outcomes. Existing studies on aPL titer fluctuations during pregnancy have only focused on healthy women and patients with APS or systemic lupus erythematosus (SLE), with no longitudinal studies investigating peri-pregnancy aPL changes in women with RPL. This study aims to test serial quantification of IgA, IgM, and IgG isotypes targeting aCL and aβ_2_GPI in women with RPL. Furthermore, by tracking pregnancy and fetal outcomes between aPL-positive and aPL-negative subgroups, we will explore the correlation between these immune-related antibodies and pregnancy outcomes in RPL women. This research will provide insights to optimize treatment strategies and improve clinical pregnancy outcomes.

## 2 Patients and methods

### 2.1 Study population

This retrospective-prospective study enrolled pregnant women with RPL who visited the Department of Reproductive Medicine at the First Affiliated Hospital of Xi’an Jiaotong University from March 2022 to December 2023, with follow-up until pregnancy outcome. All participants were required to meet the following inclusion criteria: 1. Diagnosis of RPL per the 2017 ESHRE guideline (1), defined as two or more prior pregnancy losses. Qualifying losses included spontaneous clinical miscarriages and biochemical pregnancies (defined as positive hCG without ultrasound evidence of intrauterine pregnancy). Losses excluded from this count were ectopic pregnancies, molar pregnancies, elective terminations, and pregnancy reductions; 2. Pregnancy confirmed by serum hCG ≥25 IU/L; 3. Undergoing at least two typical APS antibody tests pre-conception and post-conception; 4. Absence of established causes of miscarriage, including parental chromosomal karyotype abnormalities, uterine anatomical abnormalities, endometrial pathology and hydrosalpinx, endocrine/metabolic disorders, confirmed immune diseases (e.g., APS, SLE, or Sjögren’s syndrome), thrombophilia. The number of pregnancy losses (PL) was recorded as the total count prior to the first APS test. A total of 100 RPL women were included in the analysis. At enrollment, none met the 2006 Sydney classification criteria (14) or the 2023 ACR/EULAR classification criteria for APS (15), as only a single-timepoint testing was performed. Written informed consent was obtained from all participants, and the study was approved by the Ethics Committee of the First Affiliated Hospital of Xi’an Jiaotong University (No. XJTU1AF2022LSK-078).

### 2.2 Sample collection

Data and serum samples were collected from enrolled pregnant women with RPL. 1. Demographic and clinical data of participants were recorded, including: maternal information: age, height, weight, body mass index (BMI), gravidity and parity, last menstrual period (LMP), and medical history, as well as paternal information: age, height, weight, BMI, smoking and alcohol consumption, and medical history. 2. Serum Collection: Peripheral blood samples were collected using standard vacuum blood collection tubes (without additives, anticoagulants, or procoagulants). After collection, samples were stored at 4 °C for 30 minutes, then centrifuged at 3500 rpm for 5 minutes to isolate serum. Serum samples were collected at three time points (1): pre-conception: prior to the current pregnancy (2); post-conception: first test result after confirmed pregnancy (gestational age <6 weeks based on LMP) (3); 3 months post-conception: test result closest to 90 days post-conception.

### 2.3 aPL testing

Fresh serum samples were tested using the automated chemiluminescence immunoassay analyzer BIO-FLASH (Werfen, Barcelona, Spain) via magnetic particle chemiluminescent immunoassay (CLIA). Test content included IgA, IgM, and IgG antibodies against cardiolipin (aCL) and β2-glycoprotein I (aβ_2_GPI). Fresh serum samples were incubated with magnetic particles coated with purified target antigens, followed by magnetic separation and washing. Subsequently, isoluminol-labeled mouse anti-human IgA/IgM/IgG antibodies were added, and after additional washing steps, a trigger solution (sodium hydroxide) was added to the reaction complex. Relative light units (RLU) were measured and converted to calibrated units (CU) using instrument-specific working curves generated by calibrated kits. According to manufacturer-defined thresholds, results were interpreted as positive (≥20 CU) or weak positive (≥8 CU). Here, the positive threshold (≥20 CU) corresponds to the 99th percentile of a normal population, which aligns with the standard ELISA cutoff for positivity (>40 U) and encompasses both medium- and high-titer ranges as defined in the 2006 Sydney classification criteria and the 2023 ACR/EULAR classification criteria for APS (14, 15).

LA was not measured in this study. This decision was based on the primary objective of investigating the dynamic changes of solid-phase assays (aCL and aβ_2_GPI) across the peri-pregnancy period, as their serial quantitative fluctuations remain underexplored. The methodological challenges of LA testing, which requires fresh plasma and specialized coagulology analysis, made it less compatible with the serial sampling design of this study.

### 2.4 Observed outcomes

The primary outcomes included live birth (delivery of a viable infant at or beyond 24 weeks of gestation), early pregnancy loss (spontaneous pregnancy loss at or before 12 weeks of gestation), late pregnancy loss (fetal demise occurring between 12 and 24 weeks of gestation), and preterm birth (live delivery occurring between 24 and 37 weeks of gestation). The analysis of pregnancy loss included spontaneous clinical pregnancy losses and biochemical pregnancies, while ectopic pregnancies, molar pregnancies, elective terminations, and embryo reductions were excluded from the analysis.

Gestational age determination followed standardized criteria: 1. Natural conception: Calculated from the first day of LMP; 2. Ovulation-monitored cycles (ultrasound-confirmed): LMP adjusted by setting the LMP date as ovulation date minus 14 days; 3. Intrauterine insemination (IUI): LMP defined as insemination date minus 14 days; 4. *In vitro* fertilization (IVF): Cleavage-stage embryo transfers (day 3) set the LMP as the transfer date minus 17 days; blastocyst transfers (day 5) set the LMP as the transfer date minus 19 days.

### 2.5 Statistical analysis

All statistical analyses were performed using IBM SPSS Statistics 27. For demographic and baseline characteristics: continuous variables were analyzed descriptively: normally distributed data (assessed via Shapiro-Wilk test) were expressed as mean ± standard deviation and compared using Student’s t-test, while non-normally distributed data were presented as median (IQR) and compared using the Mann-Whitney U test. Categorical variables were described as percentages (%) and compared using Pearson’s chi-square test, whereas ordinal variables were assessed using the Mann-Whitney U test. For primary outcomes: differences in aPL levels between groups at two timepoints were evaluated with the Wilcoxon matched-pairs signed-rank test, while comparisons across three timepoints used the Friedman test with Dunn’s *post-hoc* correction. Associations between aPL levels and pregnancy outcomes were examined through univariate and multivariate logistic regression models adjusted for covariates. All tests were two-tailed, with statistical significance defined as *p* ≤ 0.05.

## 3 Results

### 3.1 Characteristics of RPL patients

Among 100 RPL patients tested for APS, 61 (61%) were positive for at least one type of aPL in tests performed pre-conception, post-conception, or 3 months post-conception, while the remaining 39 (39%) were consistently aPL-negative across all three tests. Demographic and clinical data for aPL-positive (aPL+) and aPL-negative (aPL-) pregnant women are shown in **Table 1**. No statistically significant differences were observed between aPL+ and aPL- groups in maternal age, BMI, number of previous pregnancy losses, or presence of associated immune-related clinical manifestations. All patients denied smoking, alcohol abuse, and the occurrence of thrombosis. Paternal parameters, including age, BMI, and rates of smoking or alcohol consumption, also showed no significant differences. However, a statistically significant difference existed in the method of conception between the two groups (*p* = 0.018). In the aPL+ group, the most common methods were ovulation-monitored conception (37.7%, n=23) and natural conception (34.4%, n=21), whereas in the aPL- group, natural conception (53.8%, n=21) and IVF (33.3%, n=13) were predominant.

**Table 1:** Demographic and clinical characteristics of the study population for RPL women.

Characteristics^*^	Positive (n=61)	Negative (n=39)	*p*
Age (years ± SD)	31.87 ± 4.05	31.74 ± 3.61	0.875
BMI (kg/m^2^ ± SD)	22.32 ± 3.37	22.59 ± 3.15	0.504
Number of previous PL (n ± SD)	2.66 ± 1.12	2.79 ± 0.98	0.199
n=2	39 (63.9%)	18 (46.2%)	
n=3	11 (18.0%)	13 (33.3%)	
n=4	7 (11.5%)	5 (12.8%)	
n≥5	4 (6.5%)	3 (7.7%)	
Conception method			0.018
Natural conception	21 (34.4%)	21 (53.8%)	
Ovulation-monitored	23 (37.7%)	4 (10.3%)	
IUI	3 (4.9%)	1 (2.6%)	
IVF	14 (23.0%)	13 (33.3%)	
Immune-related clinical manifestations			
Oral Ulcers	5 (8.2%)	1 (2.6%)	0.189
Oropharyngeal Dryness	6 (9.8%)	2 (5.1%)	0.880
Atopy	3 (4.9%)	5 (12.8%)	0.550
Anemia	3 (4.9%)	4 (10.3%)	0.183
Cold Hands and Feet	9 (14.8%)	5 (12.8%)	0.724
Prone to Eczema	2 (3.3%)	0 (0.0%)	0.772
Alopecia	8 (13.1%)	5 (12.8%)	0.903
Number of clinical manifestations			0.965
n=1	11 (18.0%)	7 (17.9%)	
n=2	5 (8.2%)	3 (7.7%)	
n=3	5 (8.2%)	3 (7.7%)	
Male factors			
Age (years ± SD)	32.90 ± 4.32	33.36 ± 3.75	0.588
BMI (kg/m^2^ ± SD)	24.82 ± 2.82	24.30 ± 3.63	0.425
Smoke	19 (31.1%)	18 (46.2%)	0.130
Drink	4 (6.6%)	4 (10.3%)	0.774

^*^ Data are presented as mean ± standard deviation (SD) for age, BMI, and number of previous PL, and as number (%) for categorical characteristics.

BMI, body mass index; PL, pregnancy loss; IUI, intrauterine insemination; IVF, *in vitro* fertilization.

### 3.2 Fluctuations in aPL levels in RPL pregnant women may be influenced by pregnancy status

**Table 2** shows the qualitative and quantitative results of aPL in 100 RPL patients at three timepoints (including pre-conception, post-conception, or 3 months post-conception). Due to 8 patients not undergoing testing at 3 months post-conception and 34 patients experiencing early miscarriage (3 of whom had aPL results at 3 months post-conception), only 61 samples were available for APS assessment at 3 months post-conception. Due to the small number of positive cases, weak positive and positive results will be analyzed collectively in subsequent studies.

**Table 2:** Qualitative and quantitative results of aPL tests at three time points in RPL women.

Antibodies	Pre-conception	Post-conception	3 months post-conception
Number n(%)	100 (100%)	100 (100%)	61 (61%)
Qualitative result n(weak positive/positive)			
aβ_2_GPI-IgA	0/0	0/0	0/0
aβ_2_GPI-IgG	22/1	27/0	19/0
aβ_2_GPI-IgM	0/0	1/0	1/0
aCL-IgA	1/0	1/0	2/0
aCL-IgG	7/0	18/0	7/1
aCL-IgM	0/2	2/1	1/1
Quantitative result (mean ± SD)			
aβ_2_GPI-IgA	0.221 ± 0.750	0.093 ± 0.484	0.067 ± 0.305
aβ_2_GPI-IgG	6.796 ± 3.634	6.851 ± 3.078	6.767 ± 2.444
aβ_2_GPI-IgM	1.067 ± 0.862	1.160 ± 1.637	1.318 ± 1.509
aCL-IgA	2.088 ± 1.455	2.366 ± 1.813	2.907 ± 2.532
aCL-IgG	4.443 ± 2.540	5.305 ± 3.074	5.659 ± 3.359
aCL-IgM	3.187 ± 4.525	2.992 ± 3.994	3.179 ± 4.820

aβ_2_GPI, anti-β_2_-glycoprotein I antibody; aCL, anticardiolipin antibody.

**Figure 1:**
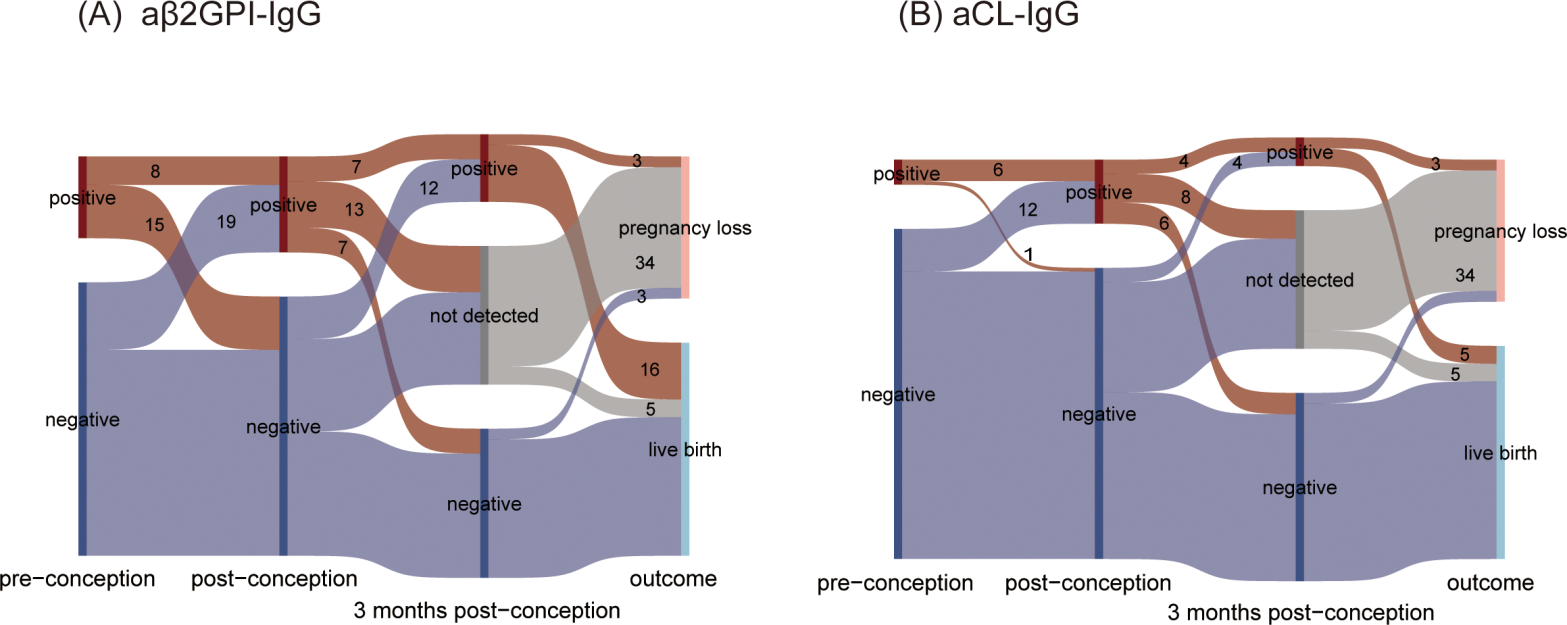
Longitudinal trajectories of specific aPL in relation to pregnancy outcomes, visualized by Sankey diagram. **(A)** aβ_2_GPI-IgG; **(B)** aCL-IgG. The aPL status (positive, negative, and not detected) and pregnancy outcomes (pregnancy loss and live birth) are represented by different colors, with numbers indicating the count of individuals.

Before conception, 29 patients already tested positive for one or two types of aPL. The positive rates of aβ_2_GPI-IgG, aCL-IgG, and aCL-IgM all increased after conception and decreased at 3 months post-conception. The most noteworthy finding concerns the antibody with the highest positivity rate. Among 23 patients who were aβ_2_GPI-IgG positive at the beginning, 15 became negative after conception, but 4 of them tested positive again at the 3rd timepoint. Furthermore, 19 other patients seroconverted to aβ_2_GPI-IgG positive at post-conception, and 6 of them reverted to negative at 3 months post-conception (**Figure 1A**).

Notably, aCL-IgG seropositivity showed significant changes between pre- and post-conception (7% vs 18%, *p* = 0.019). Among 7 patients who were aCL-IgG positive before conception, only one became negative after conception, while the others remained positive. Additionally, 12 patients seroconverted to aCL-IgG positive after conception, with 6 of them reverted to negative at 3 months post-conception (**Figure 1B**). These findings suggest that the positive rate of aPL increases after conception in RPL pregnant women, while some patients return to negative aPL levels after pregnancy. The aPL levels in RPL patients exhibit dynamic fluctuations, which may be influenced by pregnancy status and therapeutic interventions. It is important to note that all patients failed to meet the 2023 ACR/EULAR classification criteria for APS (15).

### 3.3 aPL levels in RPL patients showed significant variations due to changes in pregnancy status

Subsequently we conducted a differential analysis of aPL levels in RPL patients at pre-conception, post-conception, and 3 months post-conception (**Table 3**), including pairwise comparisons and three-timepoint comparisons. For aβ_2_GPI-IgM, statistically significant differences were observed between pre-conception and 3 months post-conception (*p* = 0.041) and across all three timepoints (*p* = 0.004). Both aCL-IgA and aCL-IgG showed significant differences in the following comparisons: pre-conception versus post-conception (*p* = 0.018, *p* < 0.001, respectively), pre-conception versus 3 months post-conception (*p* = 0.004, *p* = 0.003, respectively), and throughout the three timepoints (*p* < 0.001, *p* = 0.003, respectively). These findings suggest that aβ_2_GPI-IgM, aCL-IgA, and aCL-IgG levels in RPL women may be influenced by pregnancy status.

**Table 3:** Analysis of differences in aPL levels over three time points in RPL women.

Antibodies	Pre-conception vs post-conception		Pre-conception vs 3 months post-conception		Post-conception vs 3 months post-conception		Pre-conception vs post-conception vs 3 months post-conception	
	Z	*p*	Z	*p*	Z	*p*	χ²	*p*
aβ_2_GPI-IgA	-1.940	0.052	-1.512	0.130	-1.123	0.261	3.268	0.195
aβ_2_GPI-IgG	-0.828	0.408	-0.370	0.711	-0.506	0.613	1.154	0.562
aβ_2_GPI-IgM	-0.534	0.593	-2.043	0.041	-1.322	0.186	10.861	0.004
aCL-IgA	-2.366	0.018	-2.850	0.004	-1.605	0.108	14.933	<0.001
aCL-IgG	-3.417	<0.001	-2.960	0.003	-0.298	0.766	11.479	0.003
aCL-IgM	-1.524	0.128	-0.103	0.918	-0.050	0.960	0.164	0.921

Z, Wilcoxon matched-pairs signed rank test; χ², Friedman test.

aβ_2_GPI, anti-β_2_-glycoprotein I antibody; aCL, anticardiolipin antibody.

### 3.4 Pre-conception aPL positivity adversely affects early pregnancy outcomes

A total of 100 women with RPL were followed up to assess their pregnancy outcomes. Overall pregnancy outcomes, along with pregnancy loss stratified by gestational age (early, ≤12 weeks; late, 12–24 weeks), according to aPL status are presented in **Table 4**. Although not statistically significant, aPL+ women showed a lower live birth rate (55.7% vs 66.7%, *p* = 0.277) and a higher early pregnancy loss rate (37.7% vs 28.2%, *p* = 0.328) compared with aPL- women. However, subgroup analysis demonstrated that women who were aPL+ before pregnancy had a significantly reduced live birth rate (44.8% vs 66.2%, *p* = 0.048) and a significantly increased early pregnancy loss rate (51.7% vs 26.8%, *p* = 0.017), while no significant differences were observed in late pregnancy loss or preterm birth rates. By contrast, pregnancy outcomes showed no significant differences among women who tested aPL+ during early pregnancy. It should be noted that all aPL+ patients received targeted treatment, including LDA and heparin, with some also receiving corticosteroids (prednisone). These findings suggest an association between pre-conception aPL+ and poorer pregnancy outcomes, including significantly reduced live birth rates and increased early pregnancy loss rates. This association is consistent with a potential adverse effect on embryo implantation and early development. In contrast, newly emerged aPL+ during early pregnancy did not demonstrate significant pathogenicity.

**Table 4:** The pregnancy outcomes of aPL+ compared to aPL- in RPL pregnant women.

Outcomes	ALL			PRE			POST		
	aPL+ (n=61)	aPL-(n=39)	*p*	aPL+ (n=29)	aPL-(n=71)	*p*	aPL+ (n=51)	aPL-(n=49)	*p*
Early PL rate	23 (37.7%)	11 (28.2%)	0.328	15 (51.7%)	19 (26.8%)	0.017	17 (33.3%)	17 (34.7%)	0.886
Late PL rate	4 (6.6%)	2 (5.1%)	0.769	1 (3.4%)	5 (7.0%)	0.824	4 (7.8%)	2 (4.1%)	0.711
Premature birth rate	2 (3.3%)	5 (12.8%)	0.155	0 (0.0%)	7 (9.9%)	0.186	2 (3.9%)	5 (10.2%)	0.402
Live birth rate	34 (55.7%)	26 (66.7%)	0.277	13 (44.8%)	47 (66.2%)	0.048	30 (58.8%)	30 (61.2%)	0.806

Data are presented as number (%).

aPL, antiphospholipid antibodies; aPL+, positive for at least one type of aPL; aPL-, negative for all tested aPL types; ALL, positive for at least one type of aPL in tests performed pre-conception, post-conception, or 3 months post-conception; PRE, positive for at least one type of aPL in tests performed pre-conception only; POST, positive for at least one type of aPL in tests performed post-conception, or 3 months post-conception; PL, pregnancy loss.

It is noteworthy that all 6 patients who tested positive for aCL-IgG both pre- and post-conception experienced pregnancy loss in this pregnancy, whereas among the 12 patients who seroconverted to positive at post-conception, 7 eventually achieved a live birth (including the 6 who reverted to negative at 3 months post-conception) (**Figure 1B**).

Notably, following completion of serial aPL testing and pregnancy outcome follow-up, one participant met the 2006 Sydney classification criteria for APS (14) based on persistent aCL-IgM positivity and a history of three early pregnancy losses. However, no participants met the 2023 ACR/EULAR criteria for APS (15). Additionally, six other patients had persistent aPL positivity on repeat testing but at low titers below the classification thresholds. Among them, only one achieved a live birth, while five experienced a pregnancy loss during the study.

### 3.5 Association analysis of factors correlated with pregnancy outcomes

**Figure 2:**
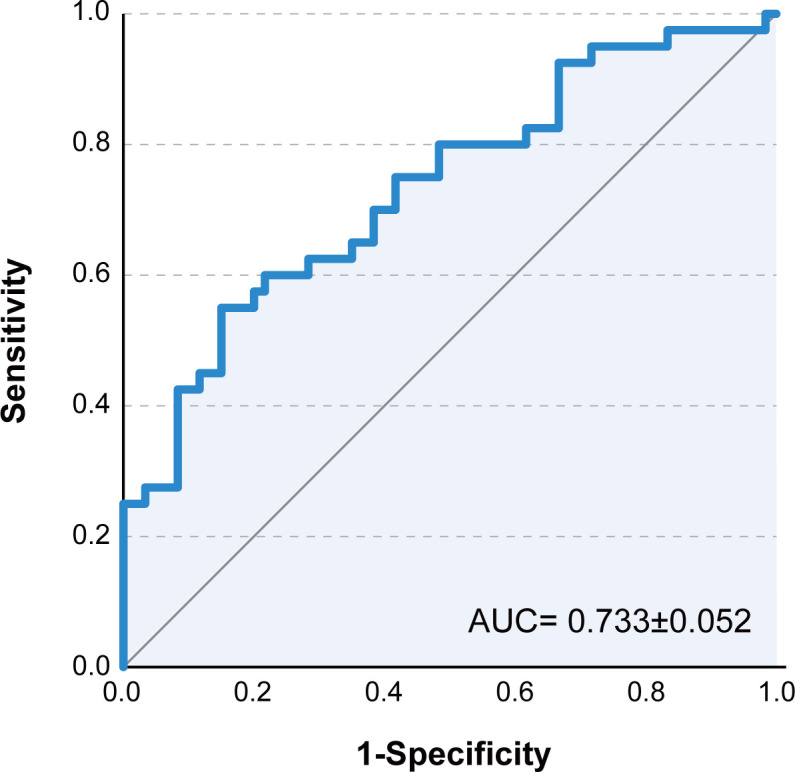
Receiver operating characteristic (ROC) curve for the logistic regression model that predicts pregnancy outcomes of RPL women. The blue line shows the model’s performance, with an area under the curve (AUC) of 0.733 (95% CI: 0.631-0.835; *p* < 0.001). The diagonal grey line represents the line of no discrimination (AUC = 0.5).

Using live birth rate as the dependent variable and relevant clinical characteristics or laboratory indicators as independent variables, univariate logistic regression analysis was first performed for each of the 43 independent variable. Variables with a p-value < 0.1 in univariate analysis were included in the multivariate logistic regression model after collinearity diagnosis, and maternal age was also included as a known predictor. The relevant results are presented in **Table 5**. After adjustment for maternal age, pre-conception aCL-IgG levels, post-conception aβ_2_GPI-IgG levels, and the number of aPL subtypes positive after conception, multivariate analysis identified the following factors as independent predictors of pregnancy loss: higher number of previous pregnancy losses (OR = 1.87, 95% CI: 1.10–3.18, *p* = 0.020), elevated post-conception aCL-IgG level (OR = 1.34, 95% CI: 1.04–1.72, *p* = 0.024), and higher number of pre-conception aPL types positive (OR = 2.73, 95% CI: 1.04–7.17, *p* = 0.042). As shown in **Figure 2**, the model demonstrated fair discrimination with an area under the receiver operating characteristic curve (AUC) of 0.733 (95% CI: 0.631-0.835, *p* < 0.001).

**Table 5:** Univariate and multivariate logistic regression analyses of pregnancy outcomes.

Variables	Univariate analysis			Multivariate analysis		
	Odds ratio	95% CI	*p*	Odds ratio	95% CI	*p*
Age^a^	1.084	0.974-1.205	0.139	0.782	0.236-2.592	0.687
Number of previous PL	1.499	0.998-2.251	0.051	1.871	1.102-3.175	0.020
aCL-IgG pre	1.213	1.021-1.441	0.028	0.909	0.687-1.203	0.504
aβ_2_GPI-IgG post	1.125	0.983-1.287	0.087	1.046	0.869-1.260	0.636
aCL-IgG post	1.258	1.079-1.465	0.003	1.336	1.039-1.718	0.024
Number of aPL+ pre	2.597	1.202-5.611	0.015	2.730	1.039-7.173	0.042
Number of aPL+ post	1.989	1.095-3.615	0.024	0.847	0.324-2.220	0.736

^a^ Age was dichotomized using a cut-off of 35 years, with the group aged <35 years serving as the reference.

post, post-conception; pre, pre-conception.

PL, pregnancy loss; aβ_2_GPI, anti-β_2_-glycoprotein I antibody; aCL, anticardiolipin antibody; aPL, antiphospholipid antibodies; aPL+, aPL positive.

## 4 Discussion

This study is the first to systematically describe the dynamic changes of aPL in the pre-conception and early pregnancy periods among RPL women. Among the 100 RPL women enrolled, 61 (61%) tested positive for at least one subtype of aPL either before conception or during early pregnancy, yet none met the 2023 ACR/EULAR classification criteria for APS (15). Significantly elevated levels of aβ_2_GPI-IgM, aCL-IgA, and aCL-IgG were observed during early pregnancy compared to pre-conception levels, and increased aCL-IgG levels at the post-conception were significantly associated with a higher risk of pregnancy loss. Analysis of pregnancy outcomes revealed that pre-conception aPL positivity was associated with a significantly reduced live birth rate and a significantly higher rate of early pregnancy loss. Additionally, the number of aPL subtypes positive at the pre-conception might be an independent risk factor for pregnancy loss. These findings suggest that aPL screening should be performed both before conception and after conception in women with RPL, enabling timely intervention to improve pregnancy outcomes.

Compared to pre-conception levels, significantly elevated quantitative levels of aCL-IgA and aCL-IgG were observed at post-conception and at 3 months post-conception in women with RPL, with a notably increased positivity rate for aCL-IgG after conception. Additionally, a significant increase in aβ_2_GPI-IgM levels was also detected at 3 months post-conception. Currently, studies on pre-conception aPL levels in RPL women remain scarce, and we found no directly comparable data. However, elevated aPL levels at post-conception are not unique to women with RPL. One study reported that healthy pregnant women also exhibited significantly higher IgG levels of aCL and aβ_2_GPI during gestation compared to non-pregnant controls (16), while another earlier study showed that pregnant women with APS had significantly higher aCL-IgG levels in early pregnancy compared to pre-conception (17). These findings are consistent with our results. The underlying mechanisms for elevated post-conception aPL levels may involve enhanced B-cell activation: the maternal immune state during pregnancy shifts from Th1 (pro-inflammatory) to Th2 (anti-inflammatory) dominance. While this helps promote fetal tolerance to maintain pregnancy (18, 19), it may disrupt immune homeostasis and activate B cells; furthermore, increased estrogen levels during pregnancy can also stimulate B-cell activation (20, 21), leading to increased production of autoantibodies including aPL. Another potential mechanism is increased exposure to self-antigens: processes such as placental formation and trophoblast invasion during pregnancy are associated with increased apoptosis (22), which may expose phospholipid-binding proteins and persistently stimulate the immune system, leading to elevated antibody levels. Simultaneously, elevated estrogen and progesterone during pregnancy promote hepatic synthesis of coagulation factors and inhibit anticoagulant pathways, resulting in a hypercoagulable state (23). Since aPL primarily contributes to thrombosis, these conditions may synergistically induce microthrombi within the placenta, causing local ischemia and tissue damage, which in turn releases more self-antigens and fuels antibody production, forming a vicious cycle. However, the precise mechanisms underlying aPL elevation and the clinical implications of changes in specific antibody subtypes require further investigation.

It is noteworthy that all aPL+ patients in this study received LDA and heparin following detection of aPL positivity at pre-conception, and a subset also received prednisone. Treatment may have directly modulated aPL levels, as immunosuppressive and anticoagulant therapies can influence immune activation and potentially reduce aPL titers (24–26). Following treatment, aPL turned negative in a subset of our patients. Consequently, the aPL levels measured after conception may not reflect the untreated biological state, leading to an underestimation of both the true aPL level and the magnitude of its fluctuation at post-conception and at 3 months post-conception in women with RPL. Therefore, the observed increases in aPL after conception may be attenuated by treatment, and that the true extent of gestational influence on aPL expression could be greater than what our data show.

During pregnancy, no significant differences were observed in levels of any aPL subtypes between the post-conception and 3 months post-conception periods. Previous studies investigating changes in aPL titers during pregnancy in healthy pregnant women or patients with APS or SLE have reported a declining trend in aPL levels throughout gestation, although these decreases lack clinical significance (17, 27–29). These findings appear inconsistent with the results of the present study. The discrepancy may be attributed to the relatively short interval between the two tests in this study (post-conception and 3 months post-conception). In contrast, previous studies compared aPL levels across the first, second, and third trimesters, with declines most commonly observed during the second trimester. Thus, differences in the timing of assessments, along with the initial establishment of maternal immune homeostasis in early pregnancy, may explain the lack of significant variation between the two time points examined in this study.

In the correlation analysis between aPL and pregnancy outcomes, this study found that pre-conception aPL positivity was a high-risk factor for early pregnancy, leading to a significantly reduced live birth rate and a significantly increased risk of early pregnancy loss. Furthermore, the number of aPL subtypes positive at the pre-conception was identified as an independent risk factor for pregnancy loss. APS is closely associated with RPL, and aPL, as its hallmark antibody, is widely recognized for its role in contributing to RPL. A 2006 meta-analysis already indicated the presence of aCL is significantly associated with early pregnancy loss (30); however, multiple studies have shown that evidence supporting a link between aβ_2_GPI and pregnancy loss remains insufficient (30–33). A systematic review highlighted that placental tissue from aPL-positive women often exhibits characteristic pathological changes, including placental infarction, impaired spiral artery remodeling, decidual inflammation, increased syncytial knots, decreased vasculosyncytial membranes, and the deposition of complement split product C4d (34). The harmful inflammatory effects of aPL on placental and endothelial cells, along with placental vascular thrombosis, may lead to placental dysfunction and subsequent pregnancy loss (3). Moreover, both the subtype and titer of aPL are associated with the risk of RPL, with higher aPL titers often correlating with more severe pregnancy complications and increased risk of pregnancy loss (26, 35).

Conversely, our study found that aPL positivity during early pregnancy did not demonstrate significant pathogenicity. This suggests that for women with RPL, pre-conception aPL testing may hold greater predictive value than testing during early pregnancy. This phenomenon may be attributed to several factors. First, aPL positivity emerging after conception may be transient; such low-titer, low-affinity antibody positivity may not possess clear pathogenic potential. In fact, aPL elevation can also occur in healthy pregnant women whose pregnancies progress uneventfully (16). Second, the most plausible explanation for the lack of significant difference in pregnancy outcomes is the efficacy of early, active intervention. All aPL+ RPL pregnant women received LDA + heparin combination therapy, a standard treatment for APS (1). Multiple studies have confirmed that this combination therapy significantly improves live birth rates and reduces the risk of pregnancy loss in aPL+ RPL patients (36–38). The success of this therapy is further evidenced by the seroconversion to negativity observed in some patients. Therefore, the comparable outcomes between aPL+ and aPL- groups should not be interpreted as evidence that aPL positivity lacks pathogenicity, but rather as a demonstration that its associated risk can be effectively mitigated by timely treatment. This treatment, while clinically essential, means that our study unavoidably underestimated the true underlying risk of aPL positivity in an untreated state. Finally, the p-value (0.048) for the pre-conception aPL+ group in this study approached the significance threshold (0.05). Considering that “positivity for any antibody at any time” itself represents a highly heterogeneous population, this finding may require validation in larger, preferably multicenter cohorts.

Among the various aPL subtypes, aCL-IgG deserves particular attention. This study found that post-conception quantitative levels of aCL-IgG significantly elevated compared to the pre-conception levels, with a marked increase in positivity rate. Multivariate regression analysis further identified post-conception aCL-IgG as an independent risk factor for pregnancy loss. A 2006 meta-analysis had already indicated a significant association between aCL presence and early pregnancy loss (30), while other studies have reported links between aCL and late pregnancy loss as well as pre-eclampsia (31, 32). Several clinical guidelines emphasize that screening for aCL is equally important as testing for LA in RPL patients, with aβ_2_GPI testing also considered as appropriate (1, 4, 9). Our findings further provide empirical support for the adverse impact of aCL positivity on pregnancy outcomes in RPL women. In addition, a history of previous pregnancy losses constitutes an independent risk factor for subsequent loss. The risk of recurrence increases significantly as the number of prior miscarriages increases. Previous studies have shown that patients with three or more consecutive pregnancy losses face a risk of subsequent miscarriage as high as 40% to 80% (4). Therefore, patients with a history of multiple pregnancy losses warrant closer clinical monitoring and management.

The evolving APS classification criteria should be noted when interpreting our findings. Only one participant met the 2006 Sydney criteria (14) following serial testing and pregnancy follow-up, and none met the 2023 ACR/EULAR criteria (15). This discrepancy reflects the fundamental difference between the two systems: the 2006 criteria prioritize sensitivity, while the 2023 criteria employ a weighted scoring system designed to maximize specificity. Importantly, six additional patients exhibited persistent low-titer aPL positivity (confirmed ≥12 weeks apart), and five of these six (83.3%) experienced pregnancy loss during the study despite not meeting formal classification. This finding suggests that RPL patients with persistent aPL positivity warrant close monitoring, as they may be at increased risk for adverse outcomes even without formal APS classification. Furthermore, our data highlight that studies using different criteria may yield different risk estimates, and direct comparisons should be made with caution. Notably, the generalizability of our results is not to the narrowly defined APS population, but to the broader RPL population that clinicians frequently need to manage without clear diagnostic guidance.

The strengths of this study include its systematic description of the dynamic changes in aPL profiles before and during pregnancy in the RPL population, which to our knowledge has not been previously reported in this patient group. All enrolled RPL women were prospectively followed for pregnancy outcomes, and aPL levels were quantified using a fully automated chemiluminescence immunoassay analyzer. However, several limitations must be acknowledged. The most important limitation is that LA was not measured. As LA is a key component of the APS classification criteria and a well-established predictor of adverse obstetric outcomes (7), its absence undoubtedly affects the comprehensiveness of our aPL assessment. This likely resulted in an underestimation of both the true aPL+ population and the strength of the observed associations. Second, the absence of an untreated comparator limits our ability to fully disentangle the natural history of aPL-related risk from the effects of therapeutic intervention. This design, while ethically necessary, introduces substantial confounding. As discussed above, the universal administration of treatment to all aPL+ patients likely led to an underestimation of the true risk associated with aPL positivity. Third, our study lacked a healthy pregnant control group. While our within-subject, longitudinal design was optimally chosen to characterize the dynamics of aPL profiles within the high-risk RPL population and to control for inter-individual variation, we acknowledge that this absence limits our ability to determine which observed changes are pathological and unique to RPL and which reflect normal pregnancy physiology. Additionally, the single-center study enhanced the internal validity of our study, even though the generalizability of our findings may be limited. Therefore, external validation in multicenter cohorts with diverse populations is warranted. Future research should involve larger sample sizes, multicenter prospective cohort studies that include healthy controls and ideally an untreated group, to further elucidate the dynamics and clinical implications of peri-pregnancy aPL profiles in RPL women.

## 5 Conclusion

This longitudinal study provides evidence that aPL profiles in women with RPL are dynamic across the peri-pregnancy period. Levels of specific aPL (including aβ_2_GPI-IgM, aCL-IgA, and aCL-IgG) were significantly elevated during pregnancy, suggesting that their expression may be influenced by gestational physiological changes. Importantly, since the number of positive aPL subtypes at pre-conception was identified as a potential independent risk factor for subsequent pregnancy loss, pre-conception aPL screening showed higher predictive value for pregnancy outcomes than first trimester testing alone. Notably, elevated post-conception aCL-IgG was identified as an independent risk factor for pregnancy loss. Therefore, even among women with RPL who test negative before conception, aPL testing in early pregnancy may facilitate early risk identification and timely initiation of standard anticoagulant and immunomodulatory therapy.

## Data Availability

The raw data supporting the conclusions of this article will be made available by the authors, without undue reservation.
